# Polymeric silk fibroin hydrogel as a conductive and multifunctional adhesive for durable skin and epidermal electronics

**DOI:** 10.1002/SMMD.20240027

**Published:** 2024-09-16

**Authors:** Fanfan Fu, Changyi Liu, Zhenlin Jiang, Qingyu Zhao, Aining Shen, Yilun Wu, Wenyi Gu

**Affiliations:** ^1^ School of Environmental and Biological Engineering Nanjing University of Science and Technology Nanjing China; ^2^ College of Chemistry and Chemical Engineering Research Center for Advanced Mirco‐ and Nano‐Fabrication Materials Shanghai University of Engineering Science Shanghai China; ^3^ Shenzhen Bay Laboratory Shenzhen Guangdong China; ^4^ College of Biotechnology and Pharmaceutical Engineering Nanjing Tech University Nanjing China; ^5^ Australian Institute of Bioengineering and Nanotechnology The University of Queensland Brisbane Queensland Australia

**Keywords:** epidermal electronics, flexible sensor, hydrogel adhesives, ionic conductive, silk fibroin

## Abstract

Silk fibroin (SF)‐based hydrogels are promising multifunctional adhesive candidates for real‐world applications in tissue engineering, implantable bioelectronics, artificial muscles, and artificial skin. However, developing conductive SF‐based hydrogels that are suitable for the micro‐physiological environment and maintain their physical and chemical properties over long periods of use remains challenging. Herein, we developed an ion‐conductive SF hydrogel composed of glycidyl methacrylate silk fibroin (SilMA) and bioionic liquid choline acylate (ChoA) polymer chains, together with the modification of acrylated thymine (ThyA) and adenine (AdeA) functional groups. The resulting polymeric ion‐conductive SF composite hydrogel demonstrated high bioactivity, strong adhesion strength, good mechanical compliance, and stretchability. The formed hydrogel network of ChoA chains can coordinate with the ionic strength in the micro‐physiological environment while maintaining the adaptive coefficient of expansion and stable mechanical properties. These features help to form a stable ion‐conducting channel for the hydrogel. Additionally, the hydrogel network modified with AdeA and ThyA, can provide a strong adhesion to the surface of a variety of substrates, including wet tissue through abundant hydrogen bonding. The biocompatible and ionic conductive SF composite hydrogels can be easily prepared and incorporated into flexible skin or epidermal sensing devices. Therefore, our polymeric SF‐based hydrogel has great potential and wide application to be an important component of many flexible electronic devices for personalized healthcare.

## Graphical abstract

We present an ion‐conducting SF composite hydrogel with the robust toughness and strong adhesion without compromising the high bioactivity. The bio‐cations surrounding the ChoA chains can provide a stable ion‐conducting channel for the whole hydrogel. The resulting composite hydrogel networks were also modified with acrylate adenine (AdeA) and acrylate thymine (ThyA), thus endowed the hydrogel (SF‐Cho/AT) with strong adhesion ability. Such an ion‐conducting SF polymeric hydrogel can be incorporated into wearable electronics for real‐time on‐body detection of various physical and electrical signals.

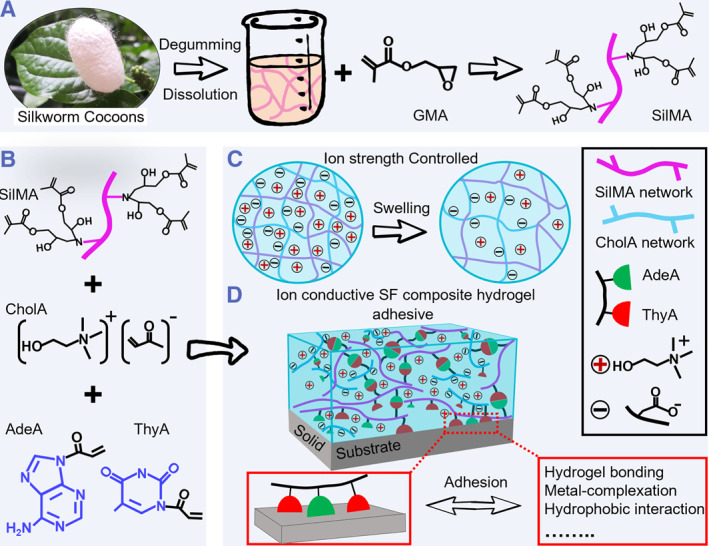

**Key points**

- We have developed a silk fibroin‐based smart interface hydrogel with super high biological activity, strong adhesion, high ionic conductivity, and controlled mechanical properties.
- The resulting smart interface hydrogel focuses on the multifunctional endowment without sacrificing the biocompatibility of silk fibroin proteins.
- The applications of such a silk fibroin‐based smart interface hydrogel in various biological interfaces are presented.

## 1 INTRODUCTION

The development of bioelectronic devices shed an ascending demand for multifunctional artificial interfaces with high biological activities, robust mechanical stabilities, and stable conductive channels.^1^, ^2^, ^3^, ^4^, ^5^, ^6^, ^7^, ^8^, ^9^, ^10^, ^11^ Currently, these artificial interface materials are mainly made of synthetic or natural polymers, and the most common forms are hydrogels and elastomers.^12^, ^13^, ^14^, ^15^ Elastomers can provide excellent mechanical properties and stability in a variety of environments but suffer from some inherent limitations like the hardness, hydrophobicity, and abiotic natures, thus are incompatible with the soft, hydrophilic, and living nature of biological tissues.^16^ Compared to elastomers, the hydrogel, as a cross‐linked polymer network with a high water content, has high biological activities and similar mechanical properties to human organs/tissues.^17^, ^18^ Additionally, hydrogels can provide a similar environment to the extracellular matrix (ECM), which is crucial for facilitating the exchange and communication of biomolecules across biological interfaces.^19^, ^20^, ^21^, ^22^, ^23^, ^24^, ^25^ These intrinsic properties make hydrogels an ideal class of materials for artificial interfaces.^26^, ^27^, ^28^, ^29^ The development of biological interface hydrogels with desired functionalities is thus of great value for bioelectronic devices.

Silk fibroin (SF)‐based hydrogels have played a leading role in biological interface material fabrication, such as those used in tissue/cell engineering, artificial muscles, implantable bioelectronics, wound dressings, and artificial skins.^30^, ^31^, ^32^, ^33^, ^34^ These biological applications benefit from the intrinsic properties of silk proteins, in particular their high biological activity, programmable biodegradation, non‐cellular cytotoxicity, and low inflammatory response.^35^, ^36^ SF‐based hydrogels can be developed by using *β*‐sheet domains as the hydrogel network crosslinkers, which originate from hydrogen bonding of SF polymer chains.^37^ These *β*‐sheet domain crosslinking points can be formed in an accelerated SF molecular motion under heating, robust vortexing, and strong sonication conditions. The resulting SF hydrogels can retain their inherent biological activities without additives. However, they may face difficulties in tailoring mechanical properties and imparting functionality. Therefore, the development of SF‐based hydrogels with robust cross‐linking networks by chemical or enzymatic modification, or with the integration of additional polymer networks and fillers, are important processes for improving the functionality of the hydrogel.^38^, ^39^, ^40^, ^41^, ^42^

The main functionalities considered for the development of SF‐based hydrogels for biological interfaces were focused on mechanical robustness, electronic conductivity, strong adhesion, and high biological activity.^43^, ^44^, ^45^ Taking advantage of the abundant active sites of the SF polymer chain, the SF‐based hydrogel can be suitably endowed with high adhesion and electrical conductivity by functional group modification and conductive element filling through chemical and physical interactions.^46^, ^47^ The SF‐based hydrogels mechanical performance (toughness and extensibility) can also be significantly improved by introducing a double/multi‐network or appropriate interactions into the existing silk hydrogel network.^48^, ^49^ Meanwhile, the introduction of additional chemicals or polymer networks also brings new challenges to SF‐based hydrogels, which may lead to a reduction in the bioactivity of the hydrogels. The development of an SF‐based hydrogel with a compromise between biological activity and other functionalities is the critical challenge for its real biological application.

**FIGURE 1:**
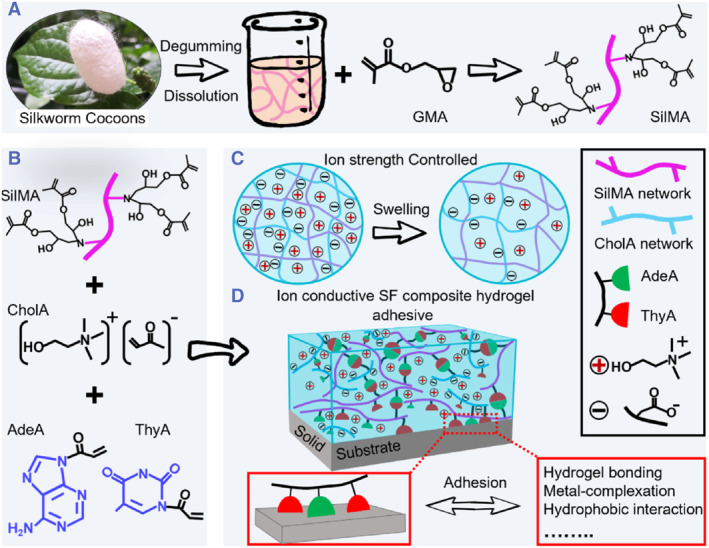
Schematic diagram of the preparation of ion‐conductive SF composite hydrogel. (A) The modification of SF with glycidyl methacrylate. (B) The chemical formula and structure of SilMA, acrylate choline (ChoA), acrylate adenine (AdeA) and acrylate thymine (ThyA). (C) Swelling diagram of ion‐conductive SF‐ChoA composite hydrogels. The swelling behavior of such composite hydrogels is controlled by the ionic strength. (D) Adhesion mechanism of ion‐conductive SF‐ChoA/AdeA‐ThyA (SF‐Cho/AT) composite hydrogel with different solid substrate surfaces.

In this work, we present an ion‐conducting SF composite hydrogel with robust toughness and strong adhesion that does not compromise the high bioactivity (Figure 1). The resulting composite hydrogel consists of glycidyl methacrylate silk fibroin (SilMA) and acylate bioionic liquid choline (ChoA) polymer chains that can regulate *β*‐sheet domains on the SF chains through electrostatic interaction, thereby improving the extensibility and toughness properties of the SF composite hydrogel. The bio‐cations surrounding the ChoA chains can provide a stable ion‐conducting channel for the whole hydrogel. Also, because of the electrostatic interaction between these bio‐cations and the negative charges of the ChoA chains, the ion‐conducting SF composite hydrogel can greatly increase the biological activity and conductivity caused by ion diffusion. In addition, the resulting composite hydrogel networks were also modified with acrylate adenine (AdeA) and acrylate thymine (ThyA), thus endowing the hydrogel (SF‐Cho/AT) with strong adhesion ability. Such an ion‐conducting SF polymeric hydrogel can be incorporated into wearable electronics for real‐time on‐body detection of various physical and electrical signals.

## 2 RESULT AND DISCUSSION

**FIGURE 2:**
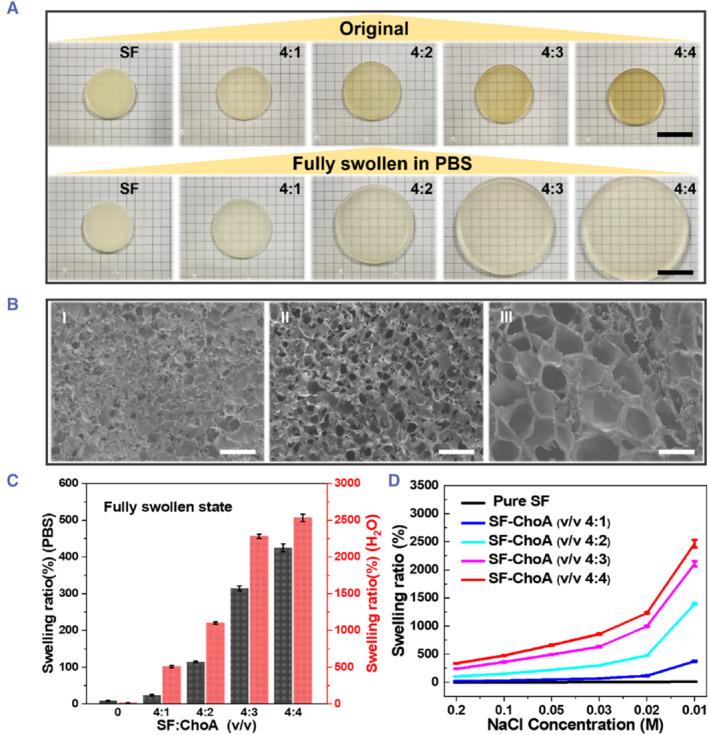
Preparation and characterization of the SF‐ChoA hydrogel. (A) Photographs of the swelling behavior (original vs. in PBS solution) of the SF‐ChoA hydrogel at different ChoA composite ratios. (B) SEM images of the SF‐ChoA hydrogel fully swollen in PBS solutions. The mixing ratio of ChoA in the hydrogel was 4:0 (I), 4:2 (II), and 4:4 (III) from left to right, respectively. (C) The swelling data (%) for the SF‐ChoA hydrogels with different ChoA ratios in PBS and Di‐water solutions. (D) The swelling data (%) for SF‐ChoA hydrogels in NaCl solution with different concentrations. Scale bars represent 2.8 cm in (A) and 200 μm in (B).

We fabricated the SF‐ChoA hydrogel with ionic strength controlled swelling ability by polymerization of SilMA and ChoA monomer to form a composite hydrogel matrix. The SilMA monomer was obtained from chemical modification of SF with glycidyl methacrylate as reported.^50^ The ChoA monomer was obtained by a chemical reaction between choline bicarbonate and acrylic acid in a molar ratio of 1:1 (Figure S1). The resulting SilMA monomer (20 wt% in water) and ChoA monomer (50 wt%, in water) were mixed and polymerized (60°C, 8 h) under different volume mixed ratios to form the SF‐ChoA hydrogels, as shown in Figure 2A. The pure SF hydrogel network can maintain almost constant volume changes in PBS solution due to the presence of chemically (glycidyl methacrylate) and physically (*β*‐sheet domains) cross‐linked points. As the number of ChoA polymers in the hydrogel increases, the swelling ratio of the SF‐ChoA hydrogel increases in the PBS solution. The determinants of the resulting hydrogel's swelling behavior are attributed to the hydrophilic properties of the ChoA chains and the electrostatic repulsion of the negatively charged (−COO^−^) groups present along the hydrogel polymer chains. As shown in Figure 2B, the swelling behavior of the hydrogel controlled by the number of ChoA polymer chains was also verified by SEM images. At a volume ratio of 4:4, the SF‐ChoA hydrogel has an apparent loose and macroporous structure, whereas the pure SF hydrogel has a dense network structure (Figure 2C‐I, III).

The SF‐ChoA hydrogels showed a higher swelling coefficient (Figure 2C) in deionized water than in PBS solution, and the swelling coefficient was also raised with increasing amounts of ChoA polymers. We believe that this difference in swelling coefficient depends on the environment of the hydrogel aqueous solution, in particular on the ionic strength and concentration.^51^, ^52^ In an electrolyte environment, the charged polymer hydrogel network of SF‐ChoA was surrounded by the electrolyte, which could affect the electrostatic repulsion between the carboxylate groups, thereby altering the swelling coefficient of the hydrogel network. The swelling coefficients of the SF‐ChoA composite hydrogels with different ChoA mixing ratios were further evaluated by using NaCl electrolyte solutions with different ionic concentrations (Figure 2D). The results also showed that the significant decrease in the swelling factor of the SF‐ChoA hydrogels could be caused by a high concentration of NaCl electrolyte solutions. In addition, the number of ChoA polymer chains also played an important role in the swelling behavior of the hydrogel networks. The water absorption capacity and the electrostatic force of the hydrogel network both increase in tandem with the increase in the proportion of ChoA polymer chains, thus influencing the swelling coefficient of the hydrogel.

**FIGURE 3:**
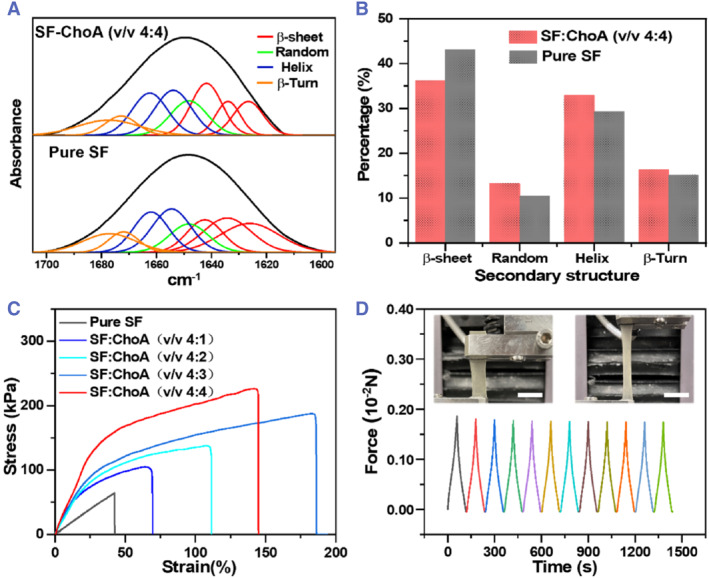
Mechanical properties of SF‐ChoA hydrogels. (A) The FTIR spectra of SF and SF‐ChoA hydrogels. (B) Quantitative analysis of the secondary structures presented in SF and SF‐ChoA hydrogels. (C) The typical stress‐strain curves of SF and SF‐ChoA hydrogels. (D) Stress‐loading/unloading curves of the SF‐ChoA hydrogel with an SF: ChoA mixed volume ratio of 4:4. Scale bars represent 1 cm in (D).

Compared to the pure silk hydrogel, the SF‐ChoA hydrogels also show an improvement in mechanical properties due to the interpenetration of the ChoA polymer networks into the SF networks (Figure 3). The molecular architectures of the SF polymer chain in SF‐ChoA hydrogels were found to be significantly different from those of SF hydrogels, as demonstrated by Fourier transform infrared spectroscopy (FTIR) in Figure 3A. From the quantitative analysis data of the secondary structures of the SF polymer network, the SF‐ChoA hydrogels presented a lower content of *β*‐sheet than the pure SF hydrogel Figure 3B. The variations in the *β*‐sheet domains, components, and numbers of the SF‐ChoA hydrogels are the main factors affecting the mechanical properties of the SF‐based hydrogels. To evaluate the mechanical properties of the SF‐ChoA hydrogels, the stress–strain curves were obtained using a mechanical tester (C42, MTS Systems Corporation). From the tensile stress‐strain test, the maximum tensile strain of the prepared pure SF hydrogel before fracture was approximately 42.3%. As the ChoA polymer network interpenetration increased, the maximum tensile strain of the prepared SF‐ChoA hydrogels (v/v 4:3) also increased to approximately 185.3%. On the other hand, as the ChoA polymer content increases to a (v/v) ratio of 4:4, the maximum tensile strain of the SF‐ChoA hydrogel decreases correspondingly (approximately 143.2%). However, the strength still increases (Figure 3C). This phenomenon in the mechanical properties is attributed to the increase in the solid content of the prepared SF‐ChoA hydrogel, which leads to the increase in the density of the hydrogel network. As the curves demonstrated in Figure 3D, we chose the SF‐ChoA hydrogel with high mechanical strength and compromise tensile strain for the stress‐loading/unloading experiment. When the SF‐ChoA hydrogel withstood 100% strain, it was able to recover quickly with a relatively small hysteresis cycle. This result indicates that the SF‐ChoA hydrogel has a good elasticity.

**FIGURE 4:**
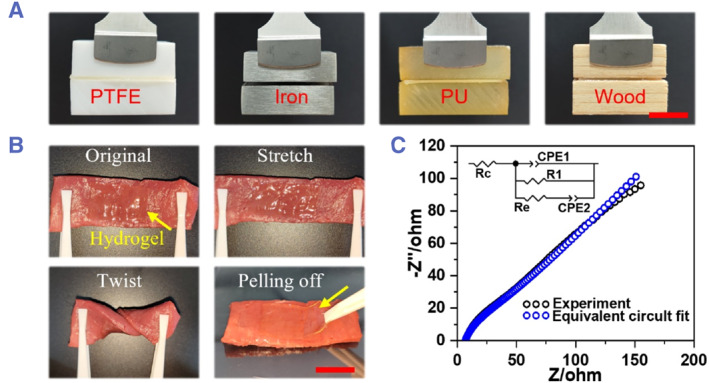
(A) Photographs of the SF‐Cho/AT composite hydrogel adhered to different substrates. (B) Photographs of the SF‐Cho/AT composite hydrogel adhered to tissue surfaces in different states. (C) Nyquist plot obtained from the electrochemical impedance spectroscopy (EIS) for the SF‐Cho/AT composite hydrogel. The inset picture is the equivalent circuit model representing the SF‐Cho/AT composite hydrogel. In the inset, the ionic resistance, electronic resistance, and total ohmic resistance of the electrochemical device are represented by the symbols Re, R1, and Rc, respectively. The constant phase elements (CPE) for the geometric capacitance and the double‐layer ionic capacitance are represented by CPEl and CPE2, respectively. Scale bars were 1.3 cm in (A), (B).

Strong adhesion to various substrate surfaces is another key feature of functional hydrogels for their application in tissue engineering, particularly for electronic devices and flexible epidermal electronics. Inspired by the coupling of double‐strand DNA molecules, the highly biocompatible nucleobases of thymine and adenine were integrated to form an SF‐Cho/AT hydrogel with adhesive interfaces. The pristine nucleobases were acrylated by reacting with acryloyl chloride to form acrylate‐adenine (AdeA) and acrylate‐thymine (ThyA) according to a report,^53^ then the resulting AdeA and ThyA were mixed with SilMA and acrylate‐choline (ChoA) to form a pregel solution. Such a pregel solution could form the SF‐Cho/AT composite hydrogel using the thermal initiator at 60°C (12 h). As shown in Figure 4A, the resulting SF‐Cho/AT hydrogel could easily adhere to polytetrafluoroethylene (PTFE), iron, polyurethane (PU), and wood. When the SF‐Cho/AT hydrogel was placed between two objects (the length, width, and height of the object were approximately 3, 2, and 1 cm) and placed vertically, the adhesion of the hydrogel was sufficient to support the weight of the objects, indicating a strong adhesive ability of the hydrogel. Moreover, the SF‐Cho/AT hydrogel adhesive can also conformably attach to porcine myocardial tissue in a variety of flexural states (Figure 4B). The strong adhesion of such a hydrogel has been attributed to the physical interactions between hydrogels and surfaces of solid materials, which are mainly dependent on hydrogen bonding and metal coordination interactions.^53^ Taking advantage of the metal coordination interactions between the SF‐Cho/AT hydrogel and the iron blocks, the hydrogel can be tightly sandwiched between two iron blocks for detection by electrochemical impedance spectroscopy (EIS). The equivalent circuit model used for the resulting hydrogels was similar to previous reports.^12^ The resulting EIS data are fitted to the equivalent circuit model shown in Figure 4C, indicating the presence of an ionic conductivity pathway in the resulting hydrogel.

**FIGURE 5:**
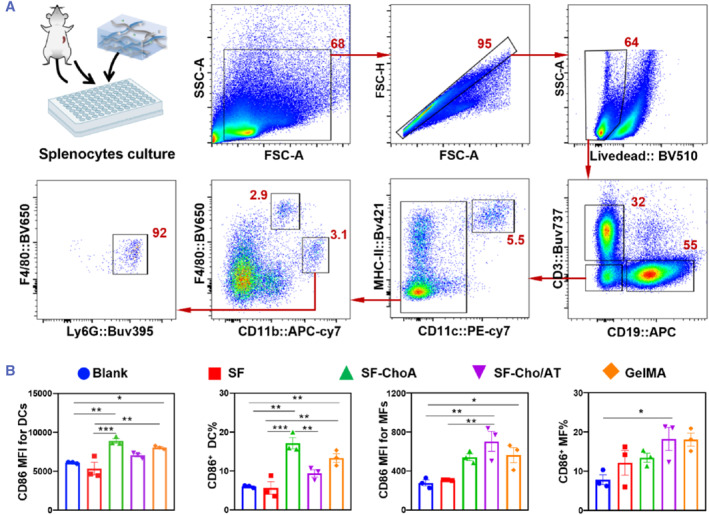
Immunogenicity and biocompatibility of SF‐hydrogels. (A) Mouse splenocyte culture and the representative flow cytometry dot plots showing the gating strategy of each cell population; (B) Histogram of the CD86 mean fluorescent intensity (MFI) and CD86 positive (CD86^+^) subset percentage of dendritic cells (DCs) and macrophages (MFs); Data are presented as the Mean ± S.D. One‐way ANOVA with Turkey's post‐test and significant signs *, *p* < 0.05; **, *p* < 0.01; ***, *p* < 0.001.

The biocompatibility and immunogenicity of SF‐Cho/AT hydrogels were also evaluated on mouse splenocytes. Immunogenicity of the resulting hydrogels was evaluated similarly to previous reports with slight modifications.^54^ Flow cytometry analysis was performed to distinguish the subsets of T lymphocytes (CD3^+^/CD19^−^), B lymphocytes (CD3^−^/CD19^+^), dendritic cells (DCs, CD11c^+^/MHC‐II^+^), macrophages (MFs, CD11b^mid^/F4/80^+^), and neutrophils (Nphs, CD11b^hi^/F4/80^−^/Ly6G^+^) using the gating F4/80 strategy shown in Figure 5A. The expression of CD86, a marker for dendritic cell (DC) maturation and M1‐like macrophage (MF) polarization, was quantified to evaluate the potency of DC‐mediated leukocyte priming and MF‐mediated inflammatory responses. SF with its well‐known good biocompatibility and the widely used GelMA were tested parallelly with our SF‐ChoA and SF‐Cho/AT for a comparison. As shown in Figure 5B, SF‐ChoA treatment led to an increased CD86 expression in DCs, resulting in 1.5‐fold of the mean fluorescent intensity (MFI) and 17% of CD86‐positive DCs compared to the blank groups. Meanwhile, SF‐Cho/AT treatment induced the highest CD86 expression in MFs among all tested materials, suggesting some M1‐like polarization. Of note, the induction of CD86 by SF‐ChoA and SF‐Cho/AT did not show statistical differences when compared to the corresponding data in GelMA groups, indicating the SF‐ChoA and SF‐Cho/AT hydrogels have a comparable immunogenicity to the GelMA. In addition, cells treated with the pristine SF did not exhibit significant influence on the DC maturation and MF polarization, in accordance with its reported low immunogenicity.^37^ Simultaneously, we calculated the percentage of live cells, T cells, B cells, MFs, DCs, and neutrophils after the culture (Figure S2). All the treatments, including blank, resulted in 65%–74% live cells, suggesting all hydrogels do not affect the cell viability in vitro. However, hydrogel variation altered the lymphocyte subset ratios. With SF‐Cho/AT, the splenocytes resulted in the highest T cell percentage (38%) and lowest B cell percentage (49%) among all treatments, whereas the T and B cell percentages in the blank group were 31% and 56%, respectively. This phenomenon may be associated with the macrophage polarization into the Th1‐dominant phenotype and release of relevant cytokines. In addition, the MFs, DCs, and Nphs manifested decreased trends in their percentages after treatments with SF‐ChoA, SF‐Cho/AT, and GelMA, whereas SF treatment did not alter these cell subsets when compared to the blank controls. Considering the increased expression of CD86, the decrease in MF and DC could be partially attributed to the maturation‐associated antigen‐presenting cell apoptosis. These changes in lymphoid and myeloid cell percentages reflected the differences in their interface bioactivities. Taken together, surface modification of SF‐ChoA and SF‐Cho/AT slightly hindered the pristine low immunogenicity of SF to endow them with conductivity and adhesive abilities. These functional hydrogels still show comparable immunogenicity to the GelMA. The H&E staining images in Figure S3 revealed that the implantation of SF, SF‐Cho/AT, and GelMA did not cause significant inflammation and leukocyte infiltration, suggesting good biocompatibility.

**FIGURE 6:**
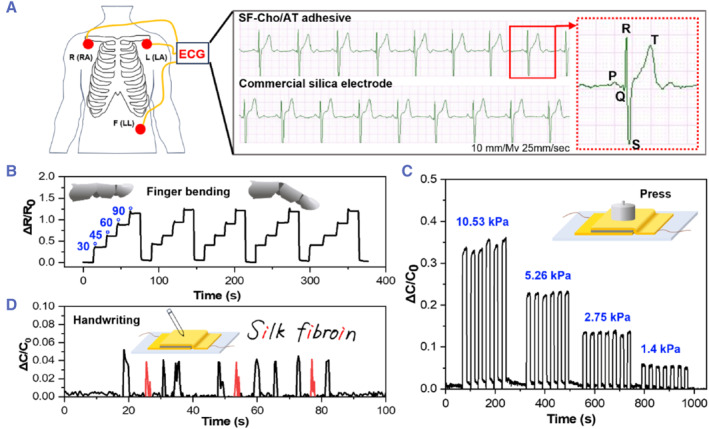
SF‐Cho/AT hydrogel applied to epidermal electronics. (A) Results of ECG signal recording from the SF‐Cho/AT adhesive hydrogel and commercial silica electrode. (B) The relative resistance change data from the bending process of the finger joint at different angles. The SF‐Cho/AT hydrogel adhered to the finger as an electronic epidermal sensor during the bending process. (C) The capacitive response of the SF‐Cho/AT hydrogel sensor system under different pressures. (D) The SF‐Cho/AT hydrogel sensor system used to monitor the writing of “Silk Fibroin”.

Based on its mechanical properties, adhesive interfaces, and good biocompatibility, the SF‐Cho/AT hydrogel adhesives can be used as an alternative electrode for recording electrocardiogram (ECG) signals. To demonstrate such an application, we used the SF‐Cho/AT hydrogel instead of the commercial silica electrode to acquire the ECG signals using the Easy ECG Monitor. As shown in Figure 6A, the SF‐Cho/AT hydrogel electrode can obtain high‐quality ECG signals that are comparable to commercial silica electrodes. Based on the two types of electrodes, the P, QRS, and T waves were identified from the recorded ECG waveforms. The acquisition of these high‐quality signals is due to the high electrical conductivity of the SF‐Cho/AT hydrogel electrode and its strong adhesion ability to the skin, which largely eliminates signal noise caused by limb movement. The SF‐Cho/AT hydrogel adhesives can also be adhered to the finger joint as a wearable strain sensor. As shown in Figure 6B, when the finger was bent to 30°, 45°, 60°, and 90° and held for 15 s (±3s), the ΔR/R_0_ value increased, and the chart data appeared in a step‐like shape. By analyzing the data collected from the five consecutive cycles, the ΔR/R_0_ value remains essentially constant when the finger is bent at the same angle. These results demonstrate the robustness and excellent reversibility of the hydrogel adhesive sensors as wearable electronic sensors.

On the other hand, the SF‐Cho/AT hydrogel can also be integrated into a capacitive strain sensor. This capacitive strain sensor was fabricated by sandwiching insulated tape VHB between two SF‐Cho/AT hydrogel electrodes that were non‐contacting and facing each other. Using this integrated sensor system, the ΔC/C_0_ value was first tested under different pressure conditions. The testing data showed that the ΔC/C_0_ value showed a significant difference under different stress conditions. From an increased pressure of 1.4–10.53 kPa, the ΔC/C_0_ value increased from 5.3% to 33.8% (Figure 6C). Taking advantage of the outstanding capacitive sensing capability, the SF‐Cho/AT hydrogel‐based capacitive pressure sensors can monitor the various pressures encountered in everyday life. As shown in Figure 6D, the capacitive response was recorded when the phrase “Silk Fibroin” was written on the surface of the integrated hydrogel sensor. The phrase “Silk Fibroin” has three identical letters “i”. The result showed that the peaks of capacitance changes produced by writing the letter “i” were highly consistent (marked with the red line in Figure 6D). The other capacitance change peaks corresponding to the other letters were also observed. These results show that different ways of writing letters can lead to different capacitance changes and form a specific peak structure. These results all indicate that the SF‐Cho/AT hydrogel sensor system has a practical value in the fields of electronic devices, strain, and pressure sensing.

## 3 CONCLUSION

In summary, we have developed a biocompatible and ion‐conductive SF‐ChoA hydrogel with controllable mechanical and swelling properties that can respond to the physiological electrolyte environment. By incorporating an additional adhesive hydrogel network (poly‐AdeA and ThyA), the resulting composite SF‐ChoA/AT hydrogel exhibits strong adhesion ability to different substrates without compromising its biocompatibility. Taking advantage of these benefits, the developed SF‐ChoA/AT hydrogel can be used to fabricate hydrogel‐based sensor systems for epidermal electronics. The sensor results indicate that such a SF‐Cho/AT hydrogel‐based sensor system is able to record the electrophysiological signals (such as ECG) and respond to joint movements (such as finger bending and handwriting traces) with a change in resistance or capacitance. Therefore, our SF‐Cho/AT hydrogel can serve as an important building block for personalized health monitoring in electronic devices, strain, and pressure sensing.

## 4 EXPERIMENTAL SECTION

### 4.1 Materials

Silkworm cocoons (bombyx mori) were obtained from Huzhou Yongrui Textile Co. Ltd. (Zhejiang Province, China). Low endotoxin GelMA, Lithium bromide (LiBr), Ammonium persulfate (APS), N, N‐dimethylformamide (DMF), Sodium Carbonate (Na_2_CO_3_), Acrylic acid (AA), diethyl ether (AR), Ammonium Persulphate, Glycidyl Methacrylate (GMA), and Choline Bicarbonate were purchased from Sigma‐Aldrich. Adenine, thymine, and acryloyl chloride were purchased from Aladdin (Shanghai, China). All other analytical reagent‐grade chemicals were commercially available. They were used without further purification. Deionized water used in all experiments was obtained from a purification system (Thermo Scientific, 18 MΩ cm).

### 4.2 Characterization

^1^H NMR spectra were obtained with an NMR spectrometer (JEOL JNM‐ECA 600) using D_2_O as solvent. FTIR measurements were performed using an FTIR spectrometer (Thermo‐Nicolet 6700). The mechanical performance of the hydrogels was measured using a mechanical tester (MTS System Corporation, C42). ECG was measured using an Easy ECG Monitor (PC‐80B, Heal Force Bio‐Meditech Holdings Limited). The corresponding software ECGDM serves as a medium for viewing and exporting data of measured ECG. The electrical effect of the samples was measured using a digital bridge (TH2830, Changzhou Tonghui Electronic Co., Ltd). The SF‐Cho/AT hydrogel was adhered to the finger of a puppet. The changing resistance of the hydrogel was recorded as the loop bent the finger multiple times. The sensitive and stable capacitive strain sensor was fabricated by sandwiching insulated tape VHB between two electrodes of SF‐Cho/AT hydrogel, which were no‐touched and opposite. The system exhibits different capacitances when subjected to different pressures. The scanning electron microscope (SEM) images of the prepared hydrogel were obtained by field‐emission SEM (FESEM, JEOL JSM‐7600F, 5 kV). Before taking the SEM images, the hydrogel has to be dried and sprayed with a layer of sputtered gold to improve image contrast.

### 4.3 Preparation of SilMA

Silk fibroin was first obtained by removing sericin from silkworm cocoons (*B. mori*). Briefly, silkworm cocoons (10 g) were boiled in a boiling Na_2_CO_3_ solution (0.02 M, 2L) for 60 min to remove the sericin. The degummed SF was then washed several times with distilled water and dried in a blast drying oven at 50°C (12 h). The regenerated silk fibrin (5 g) was then dissolved in 25 mL LiBr solution (9.3 M) at 60°C for 30 min to obtain a clear yellow liquid solution. When the SF was completely dissolved in the LiBr solution, the GMA solution (4 mL) was slowly added drop by drop to the solution, and the reaction temperature was maintained at 60°C for 5 hours. The resulting SilMA solution was purified by dialysis against DI water (8‐14 KDa) for 2 days. The pure SilMA solution was then frozen, lyophilized, and stored at −80°C.

### 4.4 Preparation of ChoA, AdeA, and ThyA

Choline acrylate (ChoA) was obtained from the reaction of choline bicarbonate and acrylate (AA).^55^ Briefly, the AA solution was added to the choline bicarbonate solution (80% in water) at a molar ratio of 1:1, and the mixed solution was reacted at 55°C for 12 h. The resulting ChoA solution was then purified by vacuum evaporation overnight at room temperature (RT). The AdeA, and ThyA were prepared as corroding to the report.^53^ To synthesize acrylate‐adenine (AdeA), Adenine (0.01 mol) and triethylamine (0.013 mol) were first dissolved in DMF solution (20 mL). The DMF solution was stirred in an ice bath for 30 min. Then, acryloyl chloride (0.012 mmol) was added to the above solution, and the reaction was maintained at RT for 6 h. Finally, to precipitate the AdeA, the reaction solution was added dropwise to the solution (diethyl ether, 300 mL). The pure product of AdeA can be obtained from purification under a vacuum environment to remove all solvents. The synthesis of acrylate‐thymine (ThyA) was similar to that of AdeA. Thymine (0.01 mol) and triethylamine (0.012 mol) were also first dissolved in 20 mL of DMF solution. After stirring the solution in an ice bath for 30 min, acryloyl chloride (0.015 mol) was added to the above solution and the reaction was also kept at RT for 6 h. Finally, the pure product of ThyA was obtained using a recrystallization and purification process similar to that of AdeA.

### 4.5 Preparation of SF‐ChoA and SF‐Cha/AT adhesive hydrogel

The SF‐ChoA hydrogel was synthesized by mixing the SilMA (20 wt% in water) and ChoA (50 wt% in water) solutions with different volume ratios. Then, thermal initiator (APS, 0.6 wt%) was also mixed with pregel solution, and the above solution was poured into the mold. The above mold was placed in an oven (60°C, 12 h) to polymerize the monomer and form the SF‐ChoA hydrogels. For the synthesis of the SF‐Cha/AT adhesive, the SilMA and ChoA solutions were also first mixed with different volume ratios. The monomer of AdeA (2 wt%) and ThyA (2 wt%), together with the thermal initiator (APS, 0.6 wt%) were then added to the pregel solution. The resulting solution was poured into the mold and polymerized in an oven (60°C, 12 h) to form the SF‐ChoA/AT hydrogels.

### 4.6 Splenocyte isolation and analysis by multi‐parameter flow cytometry

Splenocytes were obtained from 6‐8‐week‐old C57BL/6 mice. In brief, spleens were dissected and chopped into mince, followed by 1 h incubation in IMDM containing collagenase D (0.8 mg/mL, Roche, Switzerland) and DNase (40 μg/mL, Roche, Switzerland) at 37°C. Then, a single‐cell suspension was acquired by meshing against a 100 μm stainless steel strainer. Red blood cells were lysed with 0.89% ammonium chloride solution for 10 min at RT. Then, the splenocytes were centrifuged at 600 g for 5 min.

To assess the biocompatibility of hydrogels, hydrogels (ф = 5 mm, height = 2 mm) were soaked in IMDM medium containing 10% FBS and 5 ng/mL GM‐CSF overnight in a round bottom 96‐well plate and then co‐cultured with splenocytes with a density of 5 × 10^5^/well. After 24 h, the cells were collected by centrifugation. To perform multi‐parameter flow cytometry, the collected cells were incubated with 2.4G2 antibody and 1:1000 diluted Livedead® Fixable Aqua (Thermo Fisher Scientific, USA) at 4°C for 15 min in a serum free PBS solution. After washing, the cells were stained in an antibody cocktail for another 20 min with the following contents: PE‐cy7 labeled anti‐CD11c (Clone N418), BV421 labeled anti‐MHC‐II (Clone M5/114.15.2), APC‐cy7 labeled anti‐CD11b (Clone M1/70), BV650 labeled anti‐F4/80 (Clone BM8), PE labeled anti‐CD86 (Clone GL1), Buv395 labeled anti‐Ly6G (Clone 1A8), APC labeled anti‐CD19 (Clone 6D5), and Buv737 labeled anti‐CD3 (Clone 145‐2C11). All antibodies were diluted 1:600 in PBS containing 2% FBS for staining, except for anti‐MHC‐II (1:1000 dilution).

### 4.7 H&E staining

C57BL/6 mice were intraperitoneally injected with ketamine for anesthesia, and then SF, SF‐Cho/AT, and GelMA hydrogels were subcutaneously implanted in the right flank sides. After 2 weeks, the mice were euthanatized and the implanted skin with muscle was collected and processed with hematoxylin and eosin (H&E) staining for histological evaluation. The slices were visualized using a Leica optical microscope.

## AUTHOR CONTRIBUTIONS

The manuscript was written through the contributions of all authors. All authors have approved the final version of the manuscript.

## CONFLICT OF INTEREST STATEMENT

The authors declare no competing financial or other interests.

## ETHICS STATEMENT

All animal‐related protocols were approved by the Animal Care and Use Committee, Nanjing University of Science and Technology (AUCU‐NUST No. 202200158 and 2023013).

## Supporting information

Supporting Information S1
