# Supplementary material for: Polymeric silk fibroin hydrogel as a conductive and multifunctional adhesive for durable skin and epidermal electronics
Source: Smart Med. 2024 Sep 16;3(3):e20240027. doi: 10.1002/SMMD.20240027 (PMC11425052; doi:10.1002/SMMD.20240027)
Supplement: Supplementary file 1 — Supporting Information S1 [file SMMD-3-e20240027-s001.docx]

SI

Polymeric silk fibroin hydrogel as a conductive and multifunctional adhesive for durable skin & epidermal electronics

Fanfan Fu,^1#^ Changyi Liu,^1#^ Zhenlin Jiang, ^2^* Qingyu Zhao,^1^ Aining Shen,^3^ Yilun Wu,^4, 5^* and Wenyi Gu^5^*

^1^School of Environmental and Biological Engineering, Nanjing University of Science and Technology, Nanjing 210094, China

^2^College of Chemistry and Chemical Engineering, Research Center for Advanced Mirco- and Nano-Fabrication Materials, Shanghai University of Engineering Science, Shanghai 201620, China

^3^Shenzhen Bay Laboratory, Shenzhen, Guangdong, China

^4^ College of Biotechnology and Pharmaceutical Engineering, Nanjing Tech University, Nanjing 211816, China

^5^Australian Institute of Bioengineering and Nanotechnology, the University of Queensland, Brisbane 4072, Australia

^*^Correspondence:

Wenyi Gu, Australian Institute of Bioengineering and Nanotechnology, the University of Queensland, Brisbane 4072, Australia; Email: w.gu@uq.edu.au

Yilun Wu, College of Biotechnology and Pharmaceutical Engineering, Nanjing Tech University, 211816, Nanjing, China; Australian Institute of Bioengineering and Nanotechnology, the University of Queensland, Brisbane 4072, Australia; E-mail: [yilun.wu@njtech.edu.cn](mailto:yilun.wu@njtech.edu.cn);

Zhenlin Jiang, College of Chemistry and Chemical Engineering, Research Center for Advanced Mirco- and Nano-Fabrication Materials, Shanghai University of Engineering Science, Shanghai 201620, China; Email: [jiangzhenlin@sues.edu.cn](mailto:jiangzhenlin@sues.edu.cn)

**^#^**These authors contributed equally.


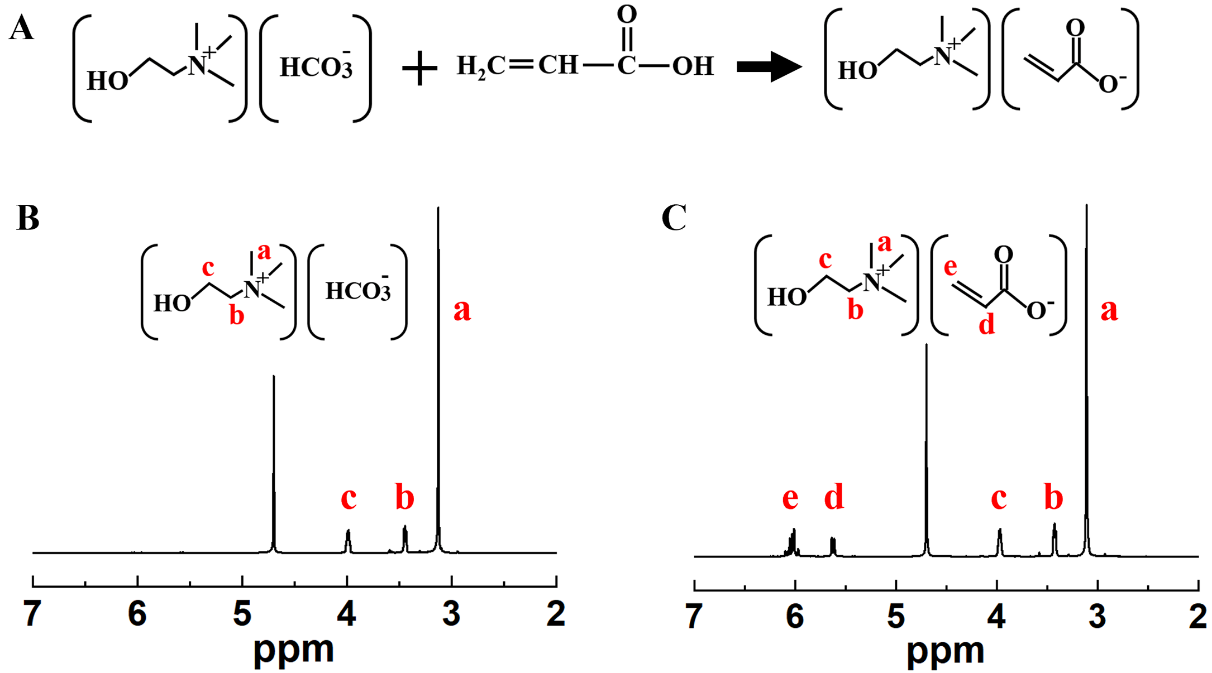


**Figure S1**. Schematic illustration of the preparation of (A) bioionic liquid choline acylate (ChoA) polymer chains. HNMR spectra of the (B ) Choline Bicarbonate and (C) ChoA.


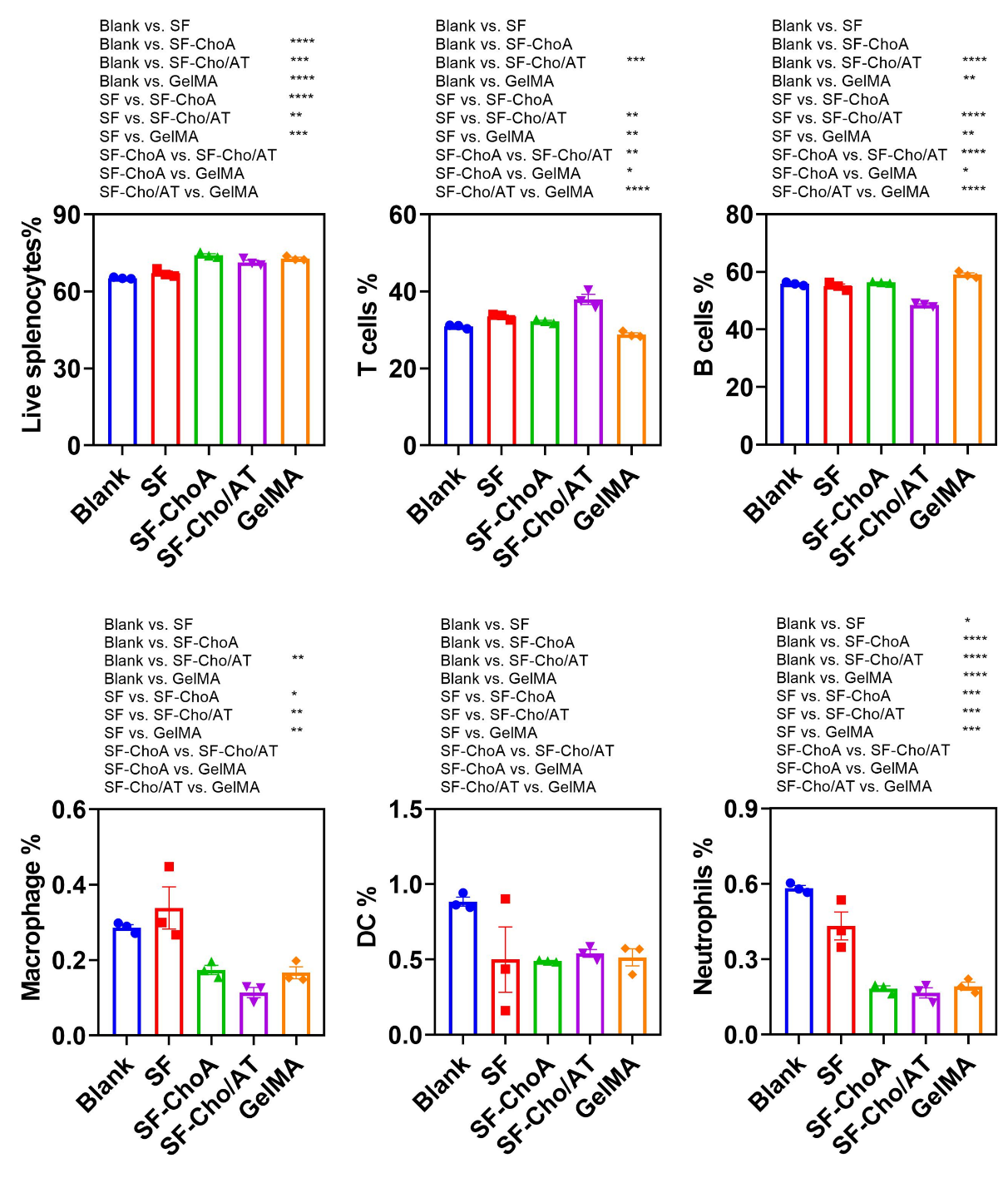


**Figure S2**. Histograms of live splenocyte percentages and different subset percentages. Data are presented as the Mean±S.D. One-way ANOVA with turkey’s post-test and significant signs*, p<0.05; **, p<0.01; ***, p<0.001.


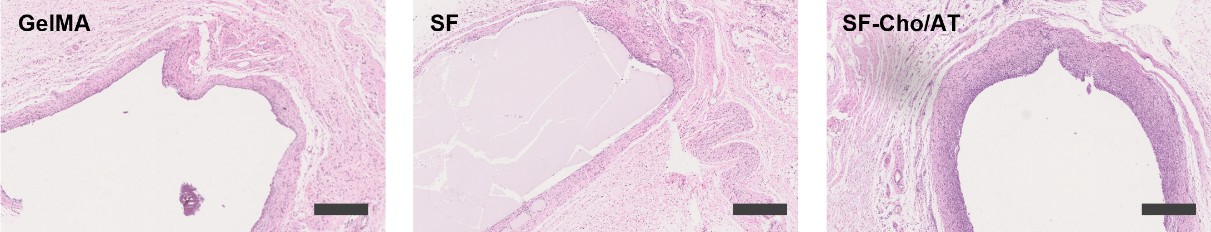


**Figure S3**. Representative H&E staining images of the mouse skin after subcutaneously hydrogel implantation. Scale bars were 500 µm.
